# Evaluating the effectiveness of reduced-intensity conditioning regimens in acute myeloid leukemia patients after hematopoietic stem cell transplantation in Vietnam

**DOI:** 10.1016/j.lrr.2026.100590

**Published:** 2026-04-22

**Authors:** Man Van Huynh, Huu Than Huynh, Thu Hanh Nguyen, Tong Thanh Tran, Xuan Tuan Ma, Nam Duy Hoang, Chi Dung Phu

**Affiliations:** a Stem Cell Transplantation Department, Blood Transfusion Hematology Hospital, Ho Chi Minh City, Vietnam; b Dong Thap General Hospital, Dong Thap, Vietnam

**Keywords:** Reduced-intensity conditioning regimens, Allogeneic transplant, Hematopoietic stem cell transplantation, Acute myeloid leukemia, Vietnam

## Abstract

**Objective:**

Hematopoietic stem cell transplantation (HSCT) is an effective treatment for acute myeloid leukemia (AML) patients, leveraging cytotoxic conditioning regimens and graft-versus-leukemia effects. Reduced-intensity conditioning (RIC) regimens extend this therapy to elderly patients and those with severe comorbidities, minimizing toxicity while maintaining efficacy. The study aims to determine the result of reduced-intensity conditioning regimens for acute myeloid leukemia patients at Blood Transfusion Hematology Hospital, Vietnam.

**Methods:**

The retrospective observational study included 21 AML patients who underwent RIC from January 2021 to March 2024.

**Results:**

Median recovery times for neutropenia and thrombocytopenia were 16 and 26 days, respectively. Complications included mucositis (95.2%), febrile episodes (85.7%), CMV reactivation (83.3%), acute GVHD (23.8%), and chronic GVHD (14.3%). The 2-year disease-free survival (DFS) and overall survival (OS) rates were 61.4% and 69.3%, respectively.

**Conclusions:**

Reduced-intensity conditioning regimens are a safe and effective treatment option for elderly AML patients with comorbidities.

## 1 Background

Hematopoietic stem cell transplantation (HSCT) is a potentially curative treatment for both malignant and non-malignant hematological diseases. In malignant conditions, conditioning regimens serve two primary purposes: (1) to eliminate the recipient's immunity, allowing engraftment and preventing graft rejection, and (2) to eradicate residual malignant cells [1].

Reduced-intensity conditioning (RIC) regimens have been developed to provide transplantation opportunities for older patients and those with significant comorbidities, minimizing toxicity while achieving therapeutic goals. The choice of regimen intensity depends on patient-specific factors such as diagnosis, disease status, age, stem cell donor type, and comorbidities.

Acute myeloid leukemia (AML) is a common indication for HSCT. Older patients often face challenges with myeloablative (MA) conditioning due to treatment-related complications. RIC regimens, while associated with more risk factors and comorbidities, have shown comparable overall survival rates to MA regimens [2].

Additionally, compared to the MA group, the RIC group shows a lower rate of non-relapse mortality (NRM) [3]; a lower risk of infection during neutropenia; and no difference in the risk of fungal infections [4]. Complications such as acute graft-versus-host disease (aGvHD) and mucosal ulcers are lower; however, the rates of chronic graft-versus-host disease (cGvHD) and cytomegalovirus (CMV) reactivation are comparable [[5], [6], [7]].

At the Blood Transfusion Hematology Hospital (BTH), RIC regimens have been applied to AML patients over the age of 50, with severe comorbidities, undergoing second HSCT or haploidentical HSCT. However, no studies have evaluated the effectiveness and toxicity of these regimens, which is why we undertook this research.

## 2 Patients and methods

This observational study retrospectively evaluated patients who underwent peripheral blood stem cell transplantation with reduced-intensity conditioning regimens at the Stem Cell Transplantation Department of BTH from January 2021 to March 2024. The study included patients diagnosed with acute myeloid leukemia (AML) who met the selection criteria, which required full post-transplant monitoring and evaluation. Patients who discontinued follow-up or lacked post-transplant monitoring were excluded. The research employed a descriptive case series study design, with a sample size consisting of all patients who qualified during the study period. Conditioning regimens consisted of fludarabine-based reduced-intensity protocols. Fludarabine was typically administered at 30 mg/m²/day for 5 days. Busulfan was given at a reduced dose (Bu2 or Bu-based regimens), and cyclophosphamide was administered at low doses in haploidentical transplantation settings. Thiotepa or melphalan was used in selected cases depending on donor type and patient characteristics. GVHD prophylaxis regimens varied depending on donor type and clinical setting, including post-transplant cyclophosphamide (PT-Cy) combined with mycophenolate mofetil (MMF) and tacrolimus (FK), MMF with FK, MMF with cyclosporine A (CsA), or methotrexate (MTX) with CsA. The main variables measured included time to neutrophil recovery (from transplantation to the first of three consecutive days with neutrophil count > 0.5 × 10^9^/L) and time to platelet recovery (from transplantation to the first of three consecutive days with a platelet count > 20 × 10^9^/L without platelet transfusion for seven days). Additionally, overall survival (OS) and disease-free survival (DFS) were evaluated, with OS defined as the time from transplantation until death or the study’s end and DFS defined as the time from HSCT until disease recurrence or the last follow-up. Data were collected and analyzed using SPSS version 14. Qualitative variables were expressed as percentages, and quantitative variables were presented as medians. Survival outcomes, including overall survival (OS) and disease-free survival (DFS), were assessed using the Kaplan-Meier method, with comparisons made using the two-sided log-rank test.

## 3 Results

The study recorded 21 patients, including 5 cases under 18 years old. Most patients were newly diagnosed with acute myeloid leukemia (AML), with 2 cases being secondary AML following breast cancer treatments. 4 cases had an HCT-CI score of ≥3, indicating secondary AML or accompanying liver or lung disease. The low/intermediate Disease Risk Index (DRI) accounted for 80.9 % of cases. Reduced-intensity conditioning (RIC) regimens used in haploidentical donor transplants included Flu/Bu/low-dose Cy and Flu/Bu/Thiotepa, with respective rates of 71.4 % and 4.8 %. Bu2/Flu and Flu/Mel regimens were used in matched related sibling 10/10 transplants. The median dose of CD34+ cells was 7.08 × 10^6^/kg (Table 1).

**Table 1:** Patient characteristics.

Characteristic	N ( %)
Age (median, range)	43 (1 - 60)
Male: Female (n)	13: 8
Disease status (n, %)	
+ de novo AML	19 (90.5)
+ secondary AML	2 (9.5)
Response (n, %)	
+ CR1	20 (95.2)
+ CR2	1 (4.8)
HCT-CI score (n, %)	
+ < 3	17 (80.9)
+ ≥ 3	4 (19.1)
DRI (n, %)	
+ Low - Intermediate	17 (80.9)
+ High - Very high	4 (19.1)
Transplant type (n, %)	
+ Matched related sibling 10/10	5 (23.8)
+ Haploidentical donor	16 (76.2)
Donor-recipient gender difference (n, %)	10 (47.6)
ABO compatibility between donor and recipient (n, %)	
+ Matched	12 (57.1)
+ Major incompatibility	3 (14.3)
+ Minor incompatibility	5 (23.8)
+ Bidirectional incompatibility	1 (4.8)
Conditioning regimen (n, %)	
+ Flu/Bu/low-dose Cy	15 (71,4)
+ Flu/Bu/Thiotepa	1 (4,8)
+ Flu/Mel	3 (14,3)
+ Bu2/Flu	2 (9,5)

## 4 Engraftment results

All patients experienced neutropenia (neutrophil count < 0.5 × 10^9^/L) post-transplant. Two patients died due to septic shock caused by multidrug-resistant Pseudomonas aeruginosa on days 12 and 13. Neutrophil recovery occurred in 90.5 % of patients, with a median recovery time of 16 days. Platelet recovery was observed in 85.7 % of patients, with a median recovery time of 26 days. Complete donor cell chimerism (>95 %) was achieved in all cases by day 28 ± 7 post-transplant.

## 5 Complications during transplantation

**Fig. 1:**
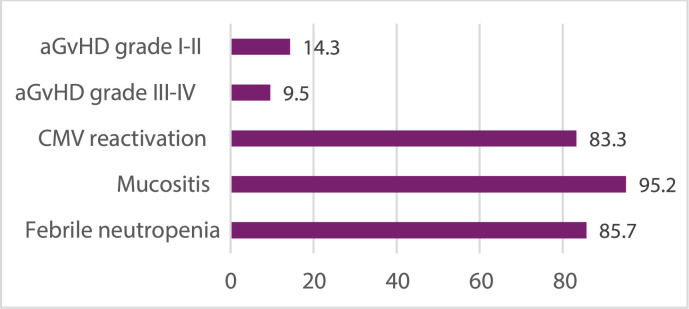
Complications within 100 days post-transplant.

Common complications included mucositis (95.2 %), febrile neutronpenia (85.7 %), and CMV reactivation (83.3 %) (Fig. 1).

All patients with mucositis required parenteral nutrition; however, none required endotracheal intubation. Broad-spectrum intravenous antibiotics were administered to 18/21 patients with febrile neutropenia, most commonly imipenem combined with vancomycin. One patient required escalation to meropenem, vancomycin, and antifungal therapy. Two patients developed severe infections requiring intensive care unit (ICU) admission and were treated with a combination of doripenem, colistin, and vancomycin.

The incidence of acute GVHD was 23.8 %, including 14.3 % grade I–II and 9.5 % grade III–IV. Chronic occurred in 14.3 % of patients, including one case (4.7 %) of extensive disease according to the modified Seattle criteria.

## 6 Outcomes after transplantation

**Fig. 2:**
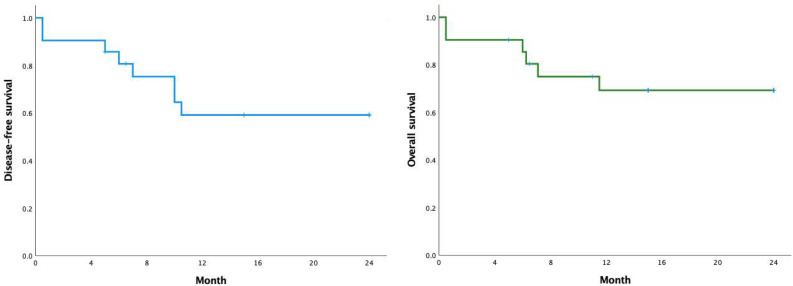
Disease-free survival and overall survival.

Patients were followed from transplantation until death or last follow-up, with a median follow-up duration of 15 months (range 0.5 - 24 months). The 6-month and 24-month disease-free survival (DFS) rates were 80.7 % and 61.4 %, respectively. The overall survival (OS) rates were 85.4 % at 6 months and 69.3 % at 24 months (Fig. 2).

When analyzing the factors affecting OS and DFS, we found no statistically significant differences between the groups: HCT-CI < 3 points and HCT-CI ≥ 3 points (*p* = 0.294), age groups < 50 years and ≥ 50 years (*p* = 0.787).

## 7 Discussion

Reduced-intensity conditioning (RIC) regimens in haploidentical hematopoietic stem cell transplantation (HSCT) with post-transplant cyclophosphamide (PT-Cy) are widely accepted for minimizing non-relapse mortality (NRM) while maintaining favorable overall survival (OS) and disease-free survival (DFS) [8,9]. At our hospital, RIC regimens are standard in haploidentical HSCT. Among 16 patients, most had low-to-intermediate Disease Risk Index (DRI) scores (13/16) and HCT-CI scores < 3 (15/16). Half-matched donors facilitated complete remission and reduced recurrence risk. Five patients had fully matched 10/10 donors, with a median age of 59 years (range 24–60), consistent with Zhou Z (median 60 years, range 18–77) [10] and Solh MM (median 63 years, range 24–75) [11]. Advances in RIC regimens have expanded HSCT eligibility to older and comorbid patients by reducing toxicity. Besides age, the presence of comorbidities, as indicated by the HCT-CI score, also influences the selection of conditioning regimens. According to Eapen M’s study, patients with an HCT-CI ≥ 3 (depending on the conditioning regimen) have a higher risk of relapse and mortality compared to those with a lower HCT-CI score [12]. Similarly, Sorror M. reported that in 391 AML patients undergoing allogeneic HSCT, 2-year survival rates varied significantly based on HCT-CI and conditioning intensity, with lower survival in high-risk groups [13].

Both age and comorbidities are crucial in conditioning regimen selection. DRI assessment before transplantation also plays a key role, as high-risk DRI correlates with increased relapse and mortality. Eapen M’s research indicated that patients with high-risk DRI had a higher incidence of relapse and mortality than those with low-risk DRI [12]. Armand P's study confirmed statistically significant differences between risk groups from low to very high (*p* < 0.0001) [14].

Chimerism rates in our study were lower than those reported by Yahng, who observed a 94 % complete chimerism (CC) rate at Day 30 in AML patients undergoing haploidentical HSCT, with the remaining 6 % achieving CC in the following month [15]. However, a cohort of 688 patients receiving HSCT with RIC reported CC rates below 90 % at Day 30 [16]. Differences across studies likely stem from variations in sample size, selection criteria, and conditioning regimens. Most patients in our study achieved engraftment, aligning with Di Stasi A’s study, which reported median engraftment times of 18 and 25 days [17]. However, our platelet engraftment times were longer. Yamashita T reported recovery times of 13 days for neutrophils and 18 days for platelets using intravenous Fludarabine/Busulfan, 14 and 18 days for oral administration, and 15 and 22 days for Fludarabine/Melphalan ^18^. The recovery times for neutrophil and platelet in Scott BL's study were 19 days and 13 days, respectively [19]; and 11 days and 10 days according to Yahng SA's study [15]. The discrepancies may be attributed to the smaller sample size in our study, predominantly involving haploidentical transplants, including one patient who had a prolonged platelet recovery time of up to 93 days, requiring eltrombopag. When compared to the study conducted by Huynh MV at BTH on a group undergoing half-matched HSCT, the grafting rates were similar, with the author reporting a grafting rate of 85.7 % and median recovery times for neutrophils and platelets of 17 (range 14–23) days and 30.5 (range 17–93) days, respectively [20]. Given that our study included some patients from Huynh’s study, result consistency is expected.

Acute graft-versus-host disease (aGvHD) rates in our study were 14.3 % (Grade I-II) and 9.5 % (Grade III-IV), comparable to Yamashita T (Grade I-II: 22.7 %; Grade III-IV: 10.6 %) [18]. A large retrospective study by the European Bone Marrow Transplantation (EBMT) group on 315 AML patients over 50 years achieving first complete remission (CR1) using RIC regimens noted significantly lower complications of Grade II-IV aGvHD (*p* = 0.01) [6]. Devine SM’s study involving 114 patients found low complication rates for Grade II-IV aGvHD at 9.6 % and for Grade III-IV at only 2.6 % [21]. The rate of chronic graft-versus-host disease (cGvHD) in our study was 14.3 %, considerably lower than in other studies, such as Yamashita T at 32.6 % [18], Scott BL at 36.9 % [19], and Yahng SA at 26.2 % [15]. These studies typically had larger sample sizes and longer follow-up periods, whereas our study had a median follow-up time of 15 months (range 0.5–24 months). A comparable study by Devine SM reported a similar cGvHD rate of 11 % in a sample of 114 patients, noting that patients who used ATG in their conditioning regimens had significantly lower rates of cGvHD (*p* = 0.002) [21].

Our 6-month and 24-month DFS rates after transplantation were comparable to those reported by Gagelmann N. in patients with myelodysplastic syndromes and by Sharma SK in AML patients [22,23]. Gagelmann N compared the efficacy of RIC and myeloablative (MA) regimens, concluding that there was no significant difference in DFS between these regimens, though no local comparisons exist at our center. However, the similarity of the outcomes of our RIC regimens to those reported globally suggests that RIC regimens are potentially effective and warrant further comparative studies in the future. Age and underlying diseases are important factors in deciding the choice of conditioning regimen and the success of the transplantation. In our study, when analyzing the correlation between age and OS and DFS, there was no significant difference among age groups. No significant OS or DFS differences emerged across age groups, consistent with McClune BL’s 10-year study (*n* = 1080), which favored RIC for older patients [24]. However, in our study, transplantation was performed on patients younger than 60 years, while those aged 60–65 typically chose conventional chemotherapy or mild treatment, indicating that detailed studies should be conducted on patients older than 60 years in the future. In contrast, Smith AR’s pediatric study showed lower 1-year OS in HCT-CI ≥ 3 groups (71 %) versus HCT-CI 0 (93 %) and 1–2 (100 %) [25], contrasting our results, possibly due to smaller sample size and fewer comorbidities in pediatric patients. In conclusion, by balancing reduced toxicity with favorable outcomes, RIC regimens expand access to potentially curative hematopoietic stem cell transplantation for a broader patient population, particularly those who have significant comorbidities.

## Ethical considerations

This study was approved by the Institutional Review Board of Blood Transfusion Hematology Hospital, Ho Chi Minh City, Vietnam, and adhered to the ethnic guidelines of Ho Chi Minh City Health department.

## Consent to participate

Patient provided written informed consent prior to participating.

## Consent for publication

Informed consent for publication was provided by the participant.

## Funding statement

No funds, grants, or other support was received.

## Data availability

The data sets generated during and/or analyzed during the current study are available from the corresponding author on reasonable request.

## CRediT authorship contribution statement

**Man Van Huynh:** Writing – review & editing, Conceptualization. **Huu Than Huynh:** Writing – review & editing, Writing – original draft, Validation, Project administration, Methodology, Investigation, Formal analysis, Data curation, Conceptualization. **Thu Hanh Nguyen:** Validation, Formal analysis, Conceptualization. **Tong Thanh Tran:** Methodology, Data curation. **Xuan Tuan Ma:** Formal analysis, Data curation. **Nam Duy Hoang:** Validation, Methodology. **Chi Dung Phu:** Supervision, Project administration.

## Declaration of competing interest

The authors declared no potential conflicts of interest with respect to the research, authorship, and/or publication of this article.
